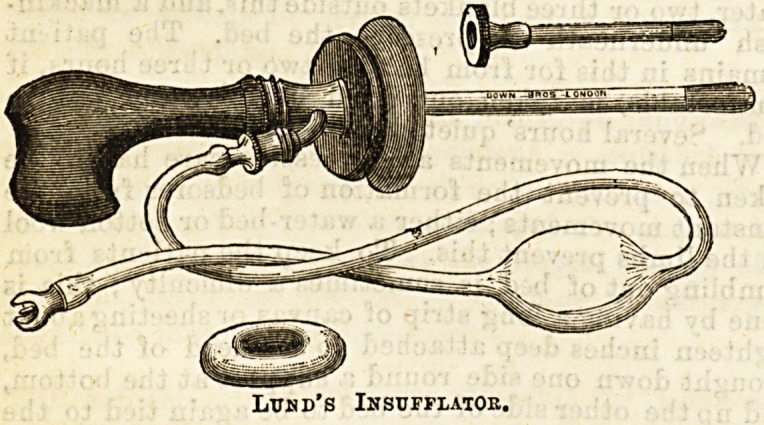# Treatment of Intussusception

**Published:** 1893-03-18

**Authors:** 


### THE HOSPITAL FOR SICK CHILDREN, GREAT ORMOND STREET.

### Treatment of Intussusception.

Success in the treatment of acute intussusception in children depends largely on the employment of prompt and energetic measures. Further, the prospects of complete and permanent relief are greatly enhanced by an early diagnosis of the condition. The treatment of acute intussusception at Great Ormond Street will be considered under the following heads: (1) The Palliative Treatment, (2) the Manipulative Treatment, (3) the Mechanical Treatment, (4) the Operative Treatment.

*The Palliative Treatment.*—This consists chiefly in the administration of opium, to which belladonna may be added. By this means pain is relieved, and peristalsis is checked, which hinders the further progress of the lesion, and may afford a possible chance of recovery. The amount of these drugs which may be prescribed will depend greatly on the urgency of the symptoms, and the age of the child.

Purgatives must on no account be given. The diet must be of a fluid kind, and the quantity given must be as small as possible.

The abdomen should be covered with warm fomentation or warm linseed poultices.

*The Manipulative Treatment.*—The child should be placed under chloroform, and sufficient should be given to render the abdominal walls perfectly lax and flaccid. The tumour must then be grasped by the two hands, and carefully kneaded and squeezed, and, as far as possible, gentle traction or rubbing should be done by one hand while the other holds the swelling.

This treatment, however, is rarely, if ever, completely successful, and is now seldom employed at Great Ormond Street, its use having been abandoned in favour of one of the two measures described under the head of mechanical treatment.

*The Mechanical Treatment.*—Under this head is included the distension of the bowel by means of air or water. Inflation is best effected by means of Lund's rectal insufflator. A Higginson's syringe may also be used, and, in cases of emergency, an ordinary pair of bellows can be made to answer the purpose. In the use of the latter instrument, the pipe of the bellows must be surrounded by a few turns of lint, secured by strapping, and then smeared with vaseline. The buttocks should be firmly compressed round the lint, and by this means the escape of air is prevented. The escape of air with the use of Lund's instrument is prevented by a rectal air pad.

The following is a diagrammatic representation of Lund's insufflator:—

Lund's Insufflator.

The child must first be placed under chloroform, and the abdominal walls rendered perfectly lax, and all efforts of straining must be completely prevented.

The tube of the apparatus is to be passed well into the rectum, and air is then slowly pumped into the colon, while the surface of the abdomen is gently manipulated. The amount of distension of the bowel can be approximately judged of by the tenseness of the abdominal parietes, and by the percussion note over the large intestine. The degree of force used must be regulated by the circumstances of the case, but it must be remembered that rupture of the bowel has occasionally resulted from this method of treatment. After the desired amount of air has been pumped into the intestine, it is well to keep it retained for a few minutes, during which time manipulation of the abdomen should be continued, and then it may be allowed to escape. The tumour must then be sought for, and if it can still be felt, the procedure must be repeated. If the tumour has disappeared, the patient may be put to bed and kept warm, and a few drops of opium administered.

The treatment of intussusception by inflation of the bowel is on the whole less popular at Gt. Ormond Street than treatment by injection of water. The reason for this is probably due to the fact that in the latter procedure the pressure within the bowel is more uniformly distributed, and consequently there is less likelihood of rupture of the intestine, and also because, in the event of rupture, the escape of sterilised water or an antiseptic solution into the peritoneal cavity would be attended with less danger to the patient than the escape of air.

Water may be injected into the bowel by means of Lund's rectal insufflator, or a Higginson's syringe. At Great Ormond Street the apparatus employed usually consists of a large glass funnel, connected by means of an ordinary piece of indiarubber piping with a vaginal tube, or a large gum elastic catheter, which is passed into the rectum. A more equable and easily regulated pressure can be obtained by this apparatus than by means of a syringe. The catheter should, before use, be passed through a cork which has been covered with a few turns of lint and smeared with vaseline, and this when fitted into the anus prevents the escape of fluid. Water previously boiled and cooled down to 100 deg., or warm boracic solution, should be employed for the injection.

The child must be placed under chloroform and the buttocks raised by means of pillows. The tube having been introduced, the amount of pressure used can be regulated by raising or depressing the funnel, which should be filled from a jug containing the injection fluid. While the water is passing into the bowel the abdominal walls should be gently manipulated, and the degree of distension should as far as possible be ascertained by means of palpation and percussion. The fluid may be withdrawn by lowering the funnel below the level of the abdomen, when the tumour should be sought for, and the operation repeated if necessary. It is hardly necessary to add that the injection of air or water into the bowel should be performed with the greatest possible care and gentleness, and no greater pressure should be used than is absolutely required. In the event of rupture of the bowel taking place, the abdomen must be opened.

*The Operative Treatment.*—If it is found impossible to reduce the intussusception by means of the treatment above described, laparotomy must be performed. This operation, however, should only be undertaken as a last resource, but if it is done at all it should be done as early as possible before the child becomes exhausted. The method of procedure is a surgical question, and need not be considered here. The operation should be performed rapidly, and the child exposed as little as possible. The after treatment must be conducted on general principles.

## Figures and Tables

**Figure f1:**